# Acetaminophen use during pregnancy and offspring attention deficit hyperactivity disorder – a longitudinal sibling control study

**DOI:** 10.1002/jcv2.12020

**Published:** 2021-06-22

**Authors:** Kristin Gustavson, Eivind Ystrom, Helga Ask, Fartein Ask Torvik, Mady Hornig, Ezra Susser, W. Ian Lipkin, Angela Lupattelli, Camilla Stoltenberg, Per Magnus, Siri Mjaaland, Ragna Bugge Askeland, Kjersti Mæhlum Walle, Michaeline Bresnahan, Hedvig Nordeng, Ted Reichborn‐Kjennerud

**Affiliations:** ^1^ Norwegian Institute of Public Health Oslo Norway; ^2^ Promenta Research Center University of Oslo Oslo Norway; ^3^ Department of Psychology University of Oslo Oslo Norway; ^4^ Center for Fertility and Health Norwegian Institute of Public Health Oslo Norway; ^5^ Mailman School of Public Health Columbia University New York NY USA; ^6^ New York State Psychiatric Institute New York NY USA; ^7^ Department of Epidemiology Columbia University Mailman School of Public Health New York NY USA; ^8^ Center for Infection and Immunity Columbia University Mailman School of Public Health New York NY USA; ^9^ Departments of Neurology and Pathology Mailman School of Public Health New York NY USA; ^10^ College of Physicians and Surgeons Columbia University New York NY USA; ^11^ Pharmacoepidemiology and Drug Safety Research Group Department of Pharmacy, and PharmaTox Strategic Research Initiative Faculty of Mathematics and Natural Sciences University of Oslo Oslo Norway; ^12^ Department of Global Public Health and Primary Care University of Bergen Bergen Norway; ^13^ Department of Medicine University of Oslo Oslo Norway

**Keywords:** acetaminophen, ADHD, MoBa, pregnancy, sibling control

## Abstract

**Background:**

Maternal acetaminophen use during pregnancy is associated with increased risk of ADHD in the child. This could reflect causal influence of acetaminophen on fetal neurodevelopment or could be due to confounding factors. The aim of the current study was to examine unmeasured familial confounding factors of this association.

**Methods:**

We used data from 26,613 children from 12,902 families participating in the prospective Norwegian Mother, Father, and Child Cohort Study (MoBa). The MoBa was linked to the Norwegian Medical Birth Register and the Norwegian Patient Registry. Siblings discordant for prenatal acetaminophen exposure were compared regarding risk of having an ADHD diagnosis.

**Results:**

Children exposed to acetaminophen up to 28 days during pregnancy did not have increased risk of receiving an ADHD diagnosis compared to unexposed children. The adjusted Hazard ratio (aHR) was 0.87 (95% C.I. = 0.70‐1.08) for exposure 1 to 7 days, and 1.13 (95% C.I. = 0.82–1.49) for 8–28 days. Long‐term exposure (29 days or more) was associated with a two‐fold increase in risk of ADHD diagnosis (aHR = 2.02, 95% C.I = 1.17–3.25). In the sibling control model, the association between long‐term acetaminophen use and ADHD in the child was aHR = 2.77 (95% C.I. = 1.48–5.05) at the between‐family level, and aHR = 1.06 (95% C.I. = 0.51–2.05) at the within‐family level.

**Conclusions:**

Both the exposed and the unexposed children of mothers with long‐term use of acetaminophen in one of the pregnancies had increased risk of receiving an ADHD diagnosis. This indicates that the observed association between long‐term acetaminophen use during pregnancy and ADHD in the child may at least partly be confounded by unobserved family factors.

## INTRODUCTION

Several studies have shown an association between maternal use of acetaminophen during pregnancy and neurodevelopmental outcomes in children, including ADHD diagnosis and symptoms (Avella‐Garcia et al., 2016; Baker et al., 2020; Brandlistuen et al., 2013; Chen et al., 2019; Liew et al., 2016; Masarwa et al., 2018; Stergiakouli et al., 2016; Thompson et al., 2014; Trønnes et al., 2019). Two previous studies using data from large birth‐cohorts linked to national health registries have reported that the association between maternal use of acetaminophen during pregnancy and ADHD diagnosis in the child was stronger (magnitude: 2‐fold increase) with longer duration of use (Liew et al., 2014; Ystrom et al., 2017). In March 2019, the European Medicines Agency concluded that epidemiological studies assessing child neurodevelopmental outcomes after prenatal exposure to acetaminophen were inconclusive (PRAC recommendations on signals, 2019).

**Key points**

- Maternal use of acetaminophen in pregnancy has been associated with increased risk of ADHD in children
- Short‐term acetaminophen use during pregnancy was not associated with ADHD diagnosis in the child
- Long‐term use was associated with a two‐fold increased risk of ADHD diagnosis
- The association between long‐term use and ADHD in the child may be due to unmeasured familial confounding factors
- Further studies need to consider unmeasured confounding factors when examining the associations between medication use during pregnancy and child outcomes
- The findings suggest that when pregnant women use acetaminophen long‐term during pregnancy, the family may have several risk factors for ADHD

Acetaminophen is commonly used during pregnancy, with prevalence rates ranging between 40% and 60% (Avella‐Garcia et al., 2016; Lupattelli et al., 2014). An in‐depth understanding of its possible effects on the fetus is therefore of great clinical importance. Biologically plausible mechanisms for effect on fetal neurodevelopment include oxidative stress and neurotoxicity (Ghanizadeh, 2012).

An alternative explanation of the association between maternal use of acetaminophen in pregnancy and child ADHD may be unmeasured confounding factors (Cooper et al., 2014; Masarwa et al., 2020). ADHD is highly heritable (Faraone & Larsson, 2019), and genetic risk of ADHD is associated with acetaminophen use during pregnancy (Leppert et al., 2019). Hence, the observed association between prenatal exposure to acetaminophen and ADHD in the child may be confounded by maternal/familial factors increasing the risk of mothers using acetaminophen during pregnancy and also affecting the child's neurodevelopment.

Sibling control studies, wherein the risk of ADHD is compared between two differently exposed siblings, may increase our understanding of potential familial confounding of the association between prenatal acetaminophen exposure and ADHD (Cooper et al., 2014; Wood et al., 2018). If there is a causal effect, the exposed sibling is expected to have a higher risk of developing ADHD than the unexposed sibling. If the association is mainly explained by familial confounding factors, the ADHD risk should be similar for the two siblings.

This study was undertaken to determine whether the association between ADHD and acetaminophen exposure reflects familial confounding factors. We employed a sibling control design, using more recent data from the same pregnancy cohort investigated in a previous study (Ystrom et al., 2017). The power to perform sibling control analyses in this cohort has increased in the last few years because the children have grown older and more children have been diagnosed with ADHD and recorded in the Norwegian Patient Registry.

## MATERIALS AND METHODS

### Sample

The sample was based on the Norwegian Mother, Father, and Child Cohort Study (MoBa) (Magnus et al., 2016; Magnus et al., 2006). MoBa is a population‐based pregnancy cohort study conducted by the Norwegian Institute of Public Health. Pregnant women from all over Norway were recruited between 1999 and 2008 when they were invited to their routine ultrasound examination in gestational week 17. The current study uses information from maternal questionnaires at gestational weeks 17 and 30, as well as 6 months after birth.

**FIGURE 1:**
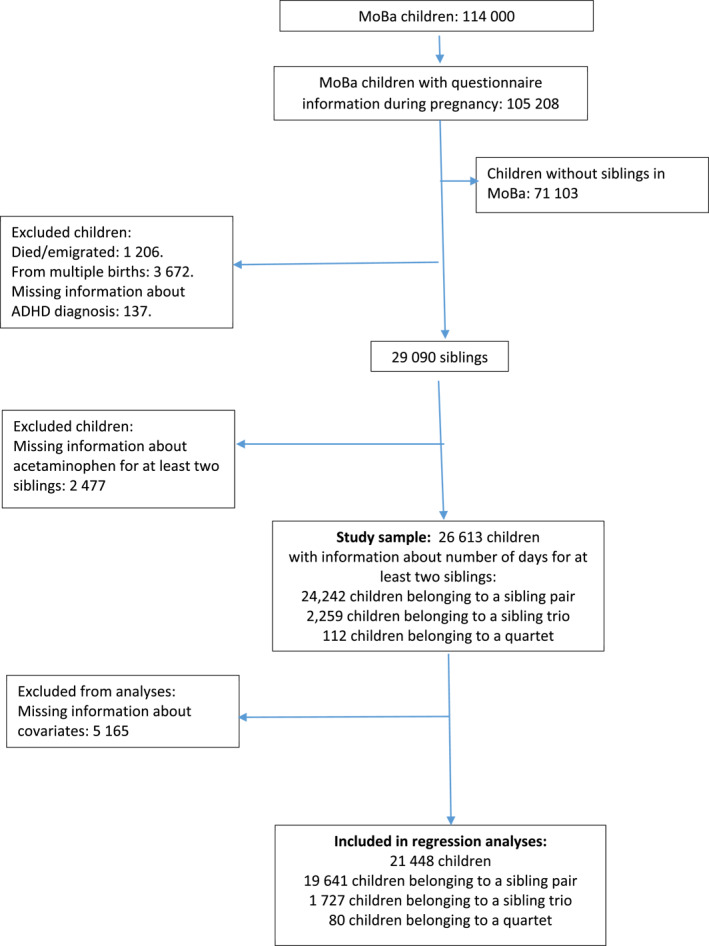
Flow chart

MoBa includes more than 114,000 children (born from 41% of the invited mothers). Approximately 30,000 of these children are siblings, and they constitute the sample of the current study. In accordance with previous studies on prenatal exposures and ADHD, children from multiple births were excluded (Dreier et al., 2016; Gustavson et al., 2017). See Figure 1 for a flow‐chart of participation, and Appendix S1 for analyses of missing data. The current study is based on version 10 of the quality‐assured MoBa data files released for research in July 2017.

### Ethical considerations

The MoBa cohort was initially licensed by the Norwegian Data protection agency and approved by The Regional Committees for Medical and Health Research Ethics, and is now regulated by the Norwegian Health Registry Act. Participants provided written consent.

### Outcome

Information about ADHD diagnoses came from the Norwegian Patient Registry (NPR). From 2008, all government‐funded clinics in Norway have reported diagnoses to the NPR. Use of privately funded mental health services for children is marginal. Children were classified as having ADHD in the current study if they were registered with Hyperkinetic disorder (F90) according to the 10th revision of the International Classification of Diseases (World Health Organization, 1993) between 2008 and 2017.

### Exposure

Mothers reported on 77, 32, and 19 different medical conditions in questionnaires at gestational weeks 17 and 30, and 6 months after birth, respectively. For each condition, the mothers reported medication use and the total number of days the medication was taken. All questionnaires had an open‐ended question about any medications used for conditions other than the ones listed. Medication use (medication name) was reported for different time‐windows within each questionnaire, while number of days was reported as a total of all time‐windows and all medications for an indication in a questionnaire. The sum of number of days' acetaminophen exposure across all indications and all questionnaires was calculated for each child.

Medications were classified according to the Anatomic Therapeutic Chemical (ATC) Classification System (WHO Collaborating Center for Drug Statistics Methodology, 2018). Acetaminophen has ATC code N02BE01. Only 1.6% of the sample was exposed to combination products including acetaminophen in addition to other active substances. See Appendix S1 for more details.

### Covariates

Information about year of birth, parity, child's sex, and maternal age was obtained from the Medical Birth Registry of Norway. Maternal educational level, smoking, alcohol use, and symptoms of depression and anxiety were reported in the MoBa questionnaire in gestational week 17. Symptoms of anxiety and depression were measured with the five item‐version of the Hopkin's symptoms checklist (Tambs & Moum, 1993).

### Statistical analyses

Propensity scores were used to adjust for measured covariates, including indications for acetaminophen use. This is a common method for reducing bias due to indication for medication use in pharmacoepidemiological studies (Glynn, 2006). Propensity scores express the estimated risk of using/being exposed to the medication in question, as predicted by observed risk factors, such as medical conditions, age, or other relevant variables. The use of propensity scores substantially reduces the number of covariates in the regression model when there are many potential confounders.

The medical conditions were classified into five different indication groups: pain conditions, fever/infections, chronic autoimmune or inflammatory conditions, unspecified conditions, and other conditions. Predicted risk (i.e., propensity) of being exposed to acetaminophen for different numbers of days (i.e., unexposed, exposed 1–7 days, 8–28 days, and 29 days or more) was calculated with the Stata “predict” option, based on the following predictors: Type and numbers of indication groups for acetaminophen use, child's birth year, maternal age, alcohol use during pregnancy, smoking during pregnancy, symptoms of anxiety and depression, use of acetaminophen before and after pregnancy, number of co‐medications used during pregnancy, and child's sex. Maternal education and parity were not included as predictors of propensity scores, as these variables were not considered appropriate covariates in the sibling control model. Maternal education is highly correlated within mothers, and parity and maternal age are highly correlated.

### Associations between acetaminophen exposure and ADHD

Cox proportional Hazard regression models were run with age at first registration of ADHD as outcome. Time at risk was defined to start with the establishment of NPR in 2008 or at age 36 months, whichever came last. The main exposure variable was number of days exposed to maternal acetaminophen use, divided into four categories (no use, use for 1–7 days, 8–28 days, and 29 days or more), with “no use” as reference. Observations were censored if they had not received an ADHD diagnosis by December 2017, when the last data from the NPR were retrieved.

First, a crude Cox regression analysis was run. Second, a Cox regression analysis was adjusted for propensity scores as well as maternal education and parity. Third, an analysis was adjusted for propensity scores and for the effects of unmeasured confounding factors shared by siblings by adding the mean of acetaminophen exposure for all siblings in a family (Begg & Parides, 2003). Dummy variables for all the exposure lengths (1–7 days, 8–28 days, and 29 days or more) were computed. The mean exposure of all siblings in a family was then calculated for each dummy.

Confidence intervals in all analyses were computed by bootstrapping (1,000 replications), to avoid assumptions of normal sampling distributions.

Sibling control analyses are prone to several sources of bias, such as lack of independence between exposure/outcome in the siblings (i.e., carryover effects), bias due to non‐shared confounding factors, and bias due to measurement error (Frisell et al., 2012; Sjolander et al., 2016). Sensitivity analyses were performed to examine these potential sources of bias. First, carryover effects were examined with a negative binominal regression analysis where number of days' exposure to acetaminophen (continuous measure) in the youngest sibling was regressed on the ADHD status of the oldest sibling, controlled for acetaminophen exposure of the oldest sibling, as suggested by Sjölander et al. (2016). Second, sensitivity analyses were performed to examine similarity between siblings regarding confounding factors. Third, as measurement error may lead to stronger attenuation of association estimates in sibling control models than in models without sibling control, sensitivity analyses were performed to examine potential effects of measurement error on the current results. This was done with the R script for simulating effects of measurement error in sibling control studies provided in Frisell et al. (2012) and adapting the script to the parameters in the current study. As described above, number of days' medication use from gestational week 30 until 6 months after birth was reported as a total of the last part of pregnancy and the months after birth if the mother had reported acetaminophen use for the same indication in both of these periods. Potential effects of measurement error due to this fact were examined in sensitivity analyses excluding acetaminophen use for indications reported both in the last part of pregnancy and in the months after birth. Potential effects of measurement error due to the fact that number of days' exposure was calculated as a total of all medications used for an indication were examined in sensitivity analyses excluding children exposed to other medications in addition to acetaminophen. More details on all of these sensitivity analyses are provided in Appendix S1.

All analyses were run in Stata version 15 (StataCorp, 2015), except the simulation analyses, which were run in RStudio version 1.4 (RStudio Team, 2020).

## RESULTS

**FIGURE 2:**
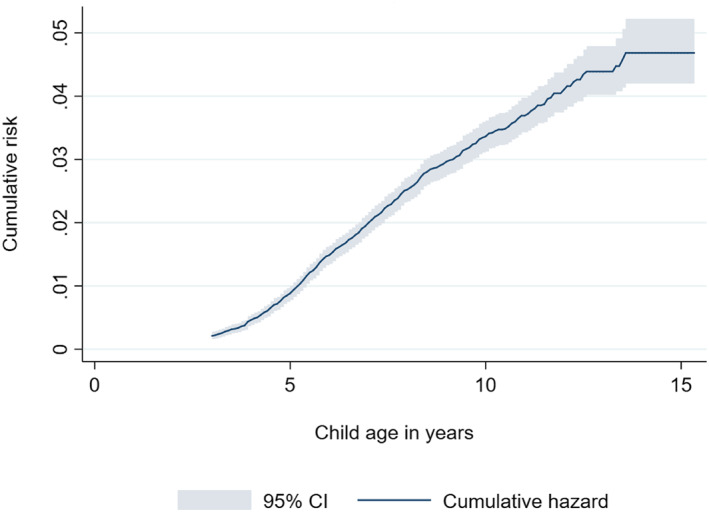
Cumulative risk of having received an ADHD diagnosis by child age

Descriptive statistics of the sample by child's exposure to acetaminophen are shown in Table 1. A total of 748 children (2.8%) received an ADHD diagnosis in the NPR. Figure 2 shows the estimated cumulative risk of ADHD by age.

**TABLE 1:** Child and parental characteristics by prenatal exposure to acetaminophen

	Unexposed *N* = 15,165 *N* (%) Median (range)	Exposed 1–7 days *N* = 7,988 *N* (%) Median (range)	Exposed 8–28 days *N* = 2,897 *N* (%) Median (range)	Exposed 29 days or more *N* = 563 *N* (%) Median (range)
Maternal age				
<25 years	1,411 (9.3%)	776 (9.7%)	237 (8.2%)	37 (6.6%)
25–34 years	11,569 (76.3%)	6,178 (77.3%)	2,223 (76.7%)	438 (77.8%)
>34 years	2,185 (14.4%)	1,034 (12.9%)	437 (15.1%)	88 (15.6%)
Missing information	0 (0%)	0 (0%)	0 (0%)	0 (0%)
Maternal education				
Less than high‐school	659 (4.4%)	340 (4.3%)	128 (4.4%)	29 (5.2%)
High‐school	3,193 (21.1%)	1,758 (22.0%)	661 (22.8%)	122 (21.7%)
University/college	10,464 (69.0%)	5,498 (68.8%)	1,977 (68.2%)	387 (68.7%)
Missing information	849 (5.6%)	392 (4.9%)	131 (4.5%)	25 (4.4%)
Maternal smoking during pregnancy				
Yes	683 (5.6%)	450 (5.6%)	155 (5.4%)	48 (8.5%)
No	13,959 (92.1%)	7,356 (92.1%)	2,695 (93.0%)	503 (89.3%)
Missing information	182 (2.3%)	182 (2.3%)	47 (1.6%)	12 (2.1%)
Maternal alcohol use during pregnancy				
Yes	1,387 (9.2%)	856 (10.7%)	334 (11.5%)	73 (13.0%)
No	11,430 (75.4%)	6,049 (75.7%)	2,208 (76.2%)	423 (75.1%)
Missing information	2,348 (15.5%)	1,083 (13.6%)	355 (12.3%)	67 (11.9%)
Maternal symptoms of depression and anxiety (scale 1–4)	1.19 (0.33)	1.21 (0.34)	1.25 (0.39)	1.31 (0.45)
Missing information	2.7%	2.1%	1.8%	1.8%
Parity				
Primiparous	6,087 (40.1%)	2,876 (36.0%)	808 (27.9%)	120 (21.3%)
Parous	9,044 (59.6%)	5,097 (63.8)	2,087 (72.0%)	443 (78.7%)
Missing information	34 (0.2%)	15 (0.2%)	2 (0.1%)	0 (0%)
Child's birth year	2005 (1999–2009)	2005 (1999–2009)	2006 (1999–2009)	2006 (2000–2009)
Missing information	0 (0.0%)	0 (0.0%)	0 (0.0%)	0 (0.0%)
Child's sex				
Boy	7,906 (52.1%)	4,066 (50.9%)	1,447 (50.0%)	278 (49.4%)
Girl	7,214 (47.6%)	3,899 (48.8%)	1,448 (50.0%)	285 (50.6%)
Missing information	45 (0.3%)	23 (0.3%)	2 (0.1%)	0 (0%)
Number of indications with pre‐pregnancy use	0 (0–2)	0 (0–3)	0 (0–3)	1 (0–3)
% yes	9.0%	31.7%	45.8%	55.6%
Number of indications with post‐pregnancy use 0–3	0 (0–0)	0 (0–2)	0 (0–3)	0 (0–4)
% yes	0%	1.7%	14.6%	43.0%
Number of indications with post‐pregnancy use 4–6	0 (0–0)	0 (0–2)	0 (0–2)	0 (0–3)
% yes	0%	1.4%	13.7%	38.4%
Number of co‐mediations		0 (0–3)	0 (0–7)	0 (0–6)
% yes		8.5%	23.3%	42.8%
ADHD diagnosis	390 (2.6%)	225 (2.8%)	104 (3.6%)	29 (5.2)

Women used acetaminophen for 75 of the 128 possible indications. Acetaminophen use for 29 days or more was reported for 48 of the indications. Pain conditions constituted 82% of the conditions for which acetaminophen was used for 29 days or more.

Siblings were discordant on exposure as well as outcome in 306 families. Siblings were discordant on exposure for 29 days or more in 380 families, and 34 of these were also discordant on the outcome. See Appendix S2 for more details on discordant siblings.

**FIGURE 3:**
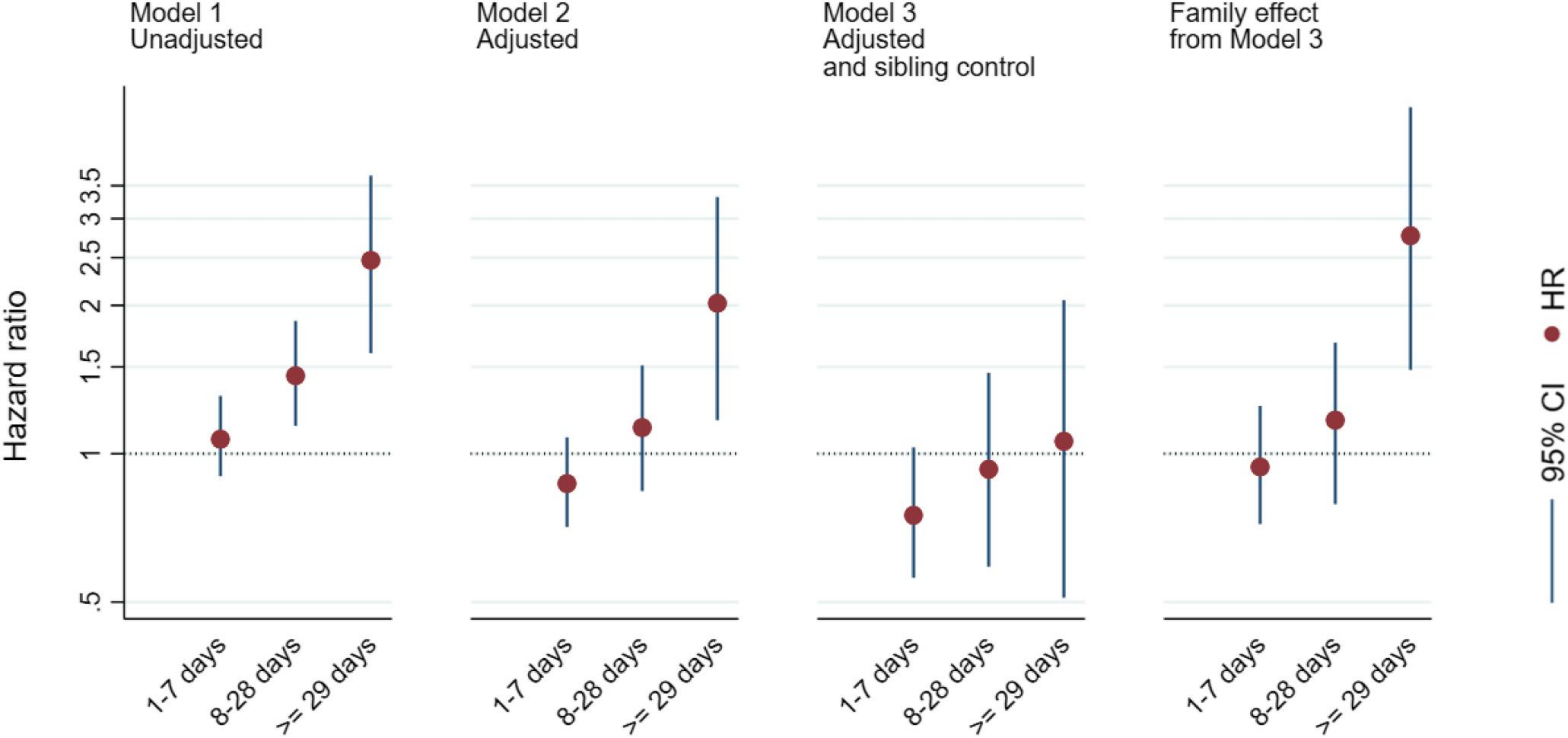
Hazard Ratios with 95% CIs by acetaminophen exposure, from three different models. *Notes:* Unexposed children are reference category in all analyses. HR, Hazard Ratio, CI, bias corrected bootstrap 95% confidence interval (1,000 replications), Model 1 is unadjusted. Model 2 is adjusted for propensity scores and maternal education and parity. Model 3 is adjusted for propensity scores. The rightmost column shows the family effect in the sibling control model

Exposure to acetaminophen for less than 29 days was not associated with increased risk of receiving an ADHD diagnosis in the adjusted model (see Table 2). Exposure for 29 days or more was associated with increased risk, with an adjusted Hazard Ratio (aHR) = 2.02 (95% bootstrap confidence interval (C.I.) = 1.17–3.25). After adjusting for the sibling mean (i.e., sibling control analyses) the association was no longer present. All children (both exposed and unexposed) born to a mother with long‐term use of acetaminophen in one pregnancy, had increased risk of receiving an ADHD diagnosis compared to children of mothers who did not use acetaminophen in any pregnancy (aHR = 2.77, 95% bootstrap C.I. = 1.48–5.05) (rightmost column in Table 2). The two leftmost columns in Figure 3 show the unadjusted and adjusted dose‐response associations between number of days of acetaminophen exposure and risk of ADHD. The third column shows that the association between long‐term exposure and ADHD was no longer present in the sibling control model. The rightmost column shows the family effect in the sibling control model.

**TABLE 2:** Cox regression results of offspring ADHD diagnosis by acetaminophen exposure during pregnancy

	Time at risk in years	Number of children	Model 1 Unadjusted	Model 2 Adjusted^a^	Model 3 Adjusted and controlled for family effect^b^	Family effect from Model 3
	(Incidence rate per 100 person years)		HR 95% C.I.	aHR 95% C.I.	aHR 95% C.I.	aHR 95% C.I.
No acetaminophen use	104,804 (0.31)	12,080	Reference	Reference	Reference	Reference
Acetaminophen 1–7 days	55,726 (0.32)	6,490	1.07	0.87	0.75	0.94
			0.90–1.31	0.70–1.08	0.56–1.03	0.72–1.25
Acetaminophen 8–28 days	20,229 (0.43)	2,409	1.44	1.13	0.93	1.17
			1.14–1.86	0.82–1.49	0.59–1.46	0.79–1.68
Acetaminophen 29 days or more	3,855 (0.73)	469	2.47	2.02	1.06	2.77
			1.60–3.67	1.17–3.25	0.51–2.05	1.48–5.05

Abbreviations: HR, Hazard Ratio; C.I., bias corrected bootstrap 95% confidence interval (1,000 replications).

The rightmost column shows the family effect in the sibling control model.

^a^ Adjusted for propensity scores and maternal education and parity.

^b^ Adjusted for propensity scores.

See text in Appendix S2 and Tables S1 and S2 in the Supporting information for results from the examination of potential bias due to missing data.

Detailed results from sensitivity analyses regarding potential bias due to carryover effects, non‐shared confounders and measurement error are presented in the Supporting information (see text in Appendix S2 as well as Tables S4 and S5 and Figures S1 and S2). In short, the results did not indicate carryover effects from ADHD in the oldest sibling to the number of days the second sibling was exposed to acetaminophen during pregnancy. Results also showed that several of the potential confounding factors were more highly correlated between siblings than were the exposure variable (i.e., being exposed to acetaminophen for 29 days or more). The results indicated some bias due to measurement error, but not enough to support the notion that measurement error was the main reason for the attenuated association estimate between long‐term acetaminophen use and ADHD in the child in the sibling control model compared to the model without sibling control.

See Table S3 in Supporting information for distribution of propensity scores in the four exposure groups.

## DISCUSSION

Children prenatally exposed to acetaminophen for 28 days or less, did not have increased risk of receiving an ADHD diagnosis, compared to unexposed children. Long‐term exposure (29 days or more) was associated with a two‐fold increase in risk. A substantial family effect in the sibling control model suggested that unmeasured familial confounding factors may explain at least part of the observed association between maternal long‐term acetaminophen use and ADHD in the child.

As discussed in the introduction, previous studies have shown an association between maternal use of acetaminophen during pregnancy and ADHD diagnosis and symptoms in children. The current results showed no evidence of increased risk of ADHD in the offspring after exposure for 28 days or less, in agreement with previous findings from the entire MoBa cohort (Ystrom et al., 2017). An association between maternal long‐term use and ADHD in the child was observed in the entire MoBa cohort (Ystrom et al., 2017) and was also present in the current study's sub‐sample of siblings. Results from the sibling control model showed that children of mothers with long‐term use in any pregnancy had increased risk of receiving an ADHD diagnosis. This family effect suggests that maternal long‐term use of acetaminophen during pregnancy may be a marker of increased familial risk for ADHD, in accordance with previous findings that mothers' genetic risk for ADHD was related to acetaminophen use during pregnancy (Leppert et al., 2019).

The association estimate for long‐term use in the sibling control model was close to 1 (i.e., no association) after controlling for the family effect discussed above. However, only discordant siblings contribute to detecting associations in sibling control models; thus, reduced power is reflected in wide confidence intervals. Our study is based on a large birth cohort and we are not aware of any other data that could be used to perform a more powered sibling control study of prenatal acetaminophen exposure and ADHD. Nevertheless, the finding of similar risk for ADHD in siblings discordant for long‐term maternal acetaminophen use must be interpreted with caution and needs to be replicated in other studies.

Previous studies have addressed the problem of unmeasured confounding factors. In one study, the association between maternal regular use of acetaminophen during pregnancy and maternally reported ADHD diagnosis in the child was compared to associations with maternal regular acetaminophen use 4 years before and 4 years after pregnancy (Liew et al., 2019). Only regular use during pregnancy was associated with ADHD, suggesting that the association could not be explained by stable confounding factors (Liew et al., 2019). Another study found that whereas maternal acetaminophen use in pregnancy increased the risk of hyperactivity symptoms in 7 year old children, paternal use during pregnancy and maternal use when the child was 5 years old did not, also suggesting that the association was not due to stable confounding factors (Stergiakouli et al., 2016). There are several potential reasons for different conclusions from different studies regarding unmeasured confounding. First, the current study focused on short‐term versus long‐term use and found that the association with long‐term use was substantially reduced in the sibling control model. Long‐term use of acetaminophen may be more strongly related to unmeasured confounding factors than short‐term use. Another reason may be that the current study used diagnoses of Hyperkinetic disorder according to the ICD‐10, with narrower inclusion criteria than the ADHD diagnosis according to DSM. Hence, the ADHD cases in the current study may be restricted to the most severe cases. A third explanation may be that while the previous studies used maternal report of diagnoses/symptoms, we used diagnoses from a national health register. Hence, our results were not affected by attrition from baseline to the time of ADHD assessment.

A previous study using the MoBa cohort found an association between maternal acetaminophen use during pregnancy and mother‐reported symptoms of externalizing symptoms when the children were three years old (Brandlistuen et al., 2013). Different results in the current study may be due to the use of ADHD diagnoses rather than maternal report of symptoms, or that children in the previous study were only three years old. Causal effects of prenatal exposure to acetaminophen may be present in very young children and then wane as the children grow older.

There are limitations that may reduce generalizability of findings. First, the response rate in MoBa was about 41% (Magnus et al., 2016), with under‐representation amongst young women, smokers, and women with a low educational level (Biele et al., 2019; Magnus et al., 2016; Nilsen et al., 2009). However, children with ADHD in MoBa are similar to children with ADHD in the entire Norwegian population regarding child global functioning and psychosocial adversity, suggesting reasonable generalizability (Oerbeck et al., 2017). Data simulation studies suggest that estimates of associations between risk factors and health outcomes are relatively robust against under‐representation of some groups in the sample (Gustavson et al., 2019). Second, as acetaminophen is an over‐the‐counter medication, we had to rely on self‐reported use of acetaminophen. Random measurement errors will generally attenuate association estimates, possibly leading to underestimation of associations. However, our finding of a more than two‐fold increased risk of ADHD among children of women who had used acetaminophen long‐term in at least one pregnancy is very similar to the association between acetaminophen use and ADHD in the child reported in a recent study that measured acetaminophen in meconium (Baker et al., 2020), suggesting that maternal report captures acetaminophen use with sufficient reliability to detect associations with child ADHD. Measurement error may attenuate association estimates more in sibling control models than in analyses without sibling control, as discussed above. This may lead to false conclusions that observed associations are due to familial confounding factors (Frisell et al., 2012). Results from several sensitivity analyses did not indicate that the attenuation of the association between long‐term acetaminophen exposure and ADHD in the sibling control model was mainly due to measurement error. Third, the sibling comparison model adjusts not only for stable confounding factors, but also for potential mediating factors that affect all siblings even if only one is exposed (Sjolander & Zetterqvist, 2017). This may lead to under‐estimation of association estimates. Fourth, missing information for some children about number of days exposed, and about covariates, may have biased results. Sensitivity analyses indicated that this bias was weak, possibly in the direction of over‐estimating the associations. Fifth, sibling control models do not control for confounders that are not shared between siblings. This may in some instances introduce bias (Frisell et al., 2012; Saunders et al., 2019). Sensitivity analyses indicated high degree of similarity between siblings regarding several potential confounders, thus supporting the use of sibling control models (Frisell et al., 2012). However, we cannot rule out that non‐shared confounding factors have introduced bias in the study. If such confounding factors were positively associated with the exposure and the outcome, associations may have been over‐estimated in the sibling control model. Sixth, results may be biased if the exposure/outcome for one sibling is dependent on the exposure/outcome of the other sibling. It is not possible to examine fully the degree to which this may be the case in a given sample. However, as described above, a sensitivity analysis of potential carryover effects from one sibling's ADHD status to the other sibling's exposure to acetaminophen did not suggest presence of such carryover effects. Seventh, having a child with ADHD may affect the decision and timing of having more children, which may have biased the current results. Eighth, the oldest children were about 8 years old when the NPR started recording individual identifiable diagnoses. Some of these children may have received an ADHD diagnosis before that. If they had not been in contact with specialist health care again between 2008 and 2017, they may appear as false negatives in the current study.

## CONCLUSION

Maternal short‐term use of acetaminophen during pregnancy was not associated with an increased risk of child ADHD diagnosis. Long‐term use was associated with a two‐fold increase in risk. Results from the sibling control analysis suggest that this association may at least partly be due to familial confounding. The results also highlight the importance of using designs that allow accounting for unmeasured confounding factors when examining prenatal risk factors for neurodevelopmental disorders. As only discordant siblings contribute to information in sibling control models, even the current very large birth cohort provided limited statistical power. Hence, the results need to be replicated in other studies.

## CONFLICT OF INTEREST STATEMENT

Eivind Ystrom is Joint Editor for JCPP *Advances*. The remaining authors have declared that they have no competing or potential conflicts of interest. [Corrections made on 22 June 2022, after first online publication: This Conflict of Interest Statement has been updated in this version.]

## AUTHORS CONTRIBUTIONS

**Gustavson** contributed to conception and design of the study, she performed the analyses, interpreted results, and drafted the initial manuscript; Ystrom had a major role in conceptualizing and designing the study, he interpreted the results, and critically reviewed the manuscript; Ask, Ask Torvik, Hornig, Susser, Lipkin, Lupattelli, Stoltenberg, Magnus, Mjaaland, Askeland, Walle, Bresnahan, Nordeng and Reichborn‐Kjennerud contributed to conception and design of the study, interpreted the results, and critically reviewed the manuscript; and all authors approved the final manuscript as submitted.

## ETHICS STATEMENT

The MoBa cohort was initially licensed by the Norwegian Data protection agency and approved by The Regional Committees for Medical and Health Research Ethics, and is now regulated by the Norwegian Health Registry Act. Participants provided written consent. [Corrections made on 22 June 2022, after first online publication: This Ethics Statement has been added in this version.]

## Supporting information

Supporting Information

## Data Availability

The data are confidential and cannot readily be shared. Researchers may apply for access to the data from The Norwegian Institute of Public Health, the national registry owners, and the Regional Committees for Medical and Health Research Ethics.

## References

[jcv212020-bib-0001] Avella‐Garcia, C. B. , Julvez, J. , Fortuny, J. , Rebordosa, C. , Garcia‐Esteban, R. , Galán, I. R. , Tardón, A. , Rodríguez‐Bernal, C. L. , Iñiguez, C. , Andiarena, A. , Santa‐Marina, L. , & Sunyer, J. (2016). Acetaminophen use in pregnancy and neurodevelopment: Attention function and autism spectrum symptoms. International Journal of Epidemiology, 45(6), 1987–1996. 10.1093/ije/dyw115 27353198

[jcv212020-bib-0002] Baker, B. H. , Lugo‐Candelas, C. , Wu, H. , Laue, H. E. , Boivin, A. , Gillet, V. , Aw, N. , Rahman, T. , Lepage, J.‐F. , Whittingstall, K. , Bellenger, J.‐P. , Posner, J. , Takser, L. , & Baccarelli, A. A. (2020). Association of prenatal acetaminophen exposure measured in meconium with risk of attention‐deficit/hyperactivity disorder mediated by frontoparietal network brain connectivity. JAMA Pediatrics, 174(11), 1073–1081. 10.1001/jamapediatrics.2020.3080 32986124 PMC7522774

[jcv212020-bib-0003] Begg, M. D. , & Parides, M. K. (2003). Separation of individual‐level and cluster‐level covariate effects in regression analysis of correlated data. Statistics in Medicine, 22(16), 2591–2602. 10.1002/sim.1524 12898546

[jcv212020-bib-0004] Biele, G. , Gustavson, K. , Czajkowski, N. O. , Nilsen, R. M. , Reichborn‐Kjennerud, T. , Magnus, P. M. , et al. (2019). Bias from self selection and loss to follow‐up in prospective cohort studies. European Journal of Epidemiology, 34(10), 927–938. 31451995 10.1007/s10654-019-00550-1

[jcv212020-bib-0005] Brandlistuen, R. E. , Ystrom, E. , Nulman, I. , Koren, G. , & Nordeng, H. (2013). Prenatal paracetamol exposure and child neurodevelopment: A sibling‐controlled cohort study. International Journal of Epidemiology, 42(6), 1702–1713. 24163279 10.1093/ije/dyt183 PMC3887567

[jcv212020-bib-0006] Chen, M.‐H. , Pan, T.‐L. , Wang, P.‐W. , Hsu, J.‐W. , Huang, K.‐L. , Su, T.‐P. , Li, C.‐T. , Lin, W.‐C. , Tsai, S.‐J. , Chen, T.‐J. , & Bai, Y.‐M. (2019). Prenatal exposure to acetaminophen and the risk of attention‐deficit/hyperactivity disorder: A nationwide study in Taiwan. Journal of Clinical Psychiatry, 80(5). 10.4088/jcp.18m12612 31509360

[jcv212020-bib-0007] Cooper, M. , Langley, K. , & Thapar, A. (2014). Antenatal acetaminophen use and attention‐deficit/hyperactivity disorder: An interesting observed association but too early to infer causality. JAMA Pediatrics, 168(4), 306–307. 10.1001/jamapediatrics.2013.5292 24566519

[jcv212020-bib-0008] Dreier, J. W. , Andersen, A.‐M. N. , Hvolby, A. , Garne, E. , Andersen, P. K. , & Berg‐Beckhoff, G. (2016). Fever and infections in pregnancy and risk of attention deficit/hyperactivity disorder in the offspring. Journal of Child Psychology and Psychiatry, 57(4), 540–548. 26530451 10.1111/jcpp.12480

[jcv212020-bib-0009] Faraone, S. V. , & Larsson, H. (2019). Genetics of attention deficit hyperactivity disorder. Molecular Psychiatry, 24(4), 562–575. 29892054 10.1038/s41380-018-0070-0 PMC6477889

[jcv212020-bib-0010] Frisell, T. , Öberg, S. , Kuja‐Halkola, R. , & Sjölander, A. (2012). Sibling comparison designs: Bias from non‐shared confounders and measurement error. Epidemiology, 23(5), 713–720. 22781362 10.1097/EDE.0b013e31825fa230

[jcv212020-bib-0011] Ghanizadeh, A. (2012). Acetaminophen may mediate oxidative stress and neurotoxicity in autism. Medical Hypotheses, 78(2), 351. 22154541 10.1016/j.mehy.2011.11.009

[jcv212020-bib-0012] Glynn, R. J. , Schneeweiss, S. , & Stürmer, T. (2006). Indications for propensity scores and review of their use in pharmacoepidemiology. Basic and Clinical Pharmacology and Toxicology, 98(3), 253–259. 16611199 10.1111/j.1742-7843.2006.pto_293.x PMC1790968

[jcv212020-bib-0013] Gustavson, K. , Roysamb, E. , & Borren, I. (2019). Preventing bias from selective non‐response in population‐based survey studies: Findings from a Monte Carlo simulation study. BMC Medical Research Methodology, 19. 10.1186/s12874-019-0757-1 PMC6567536 31195998

[jcv212020-bib-0014] Gustavson, K. , Ystrom, E. , Stoltenberg, C. , Susser, E. , Surén, P. , Magnus, P. , Knudsen, G. P. , Davey Smith, G. , Langley, K. , Rutter, M. , Aase, H. , & Reichborn-Kjennerud, T. (2017). Smoking in Pregnancy and Child ADHD. Pediatrics, 139(2), e20162509. 10.1542/peds.2016-2509 PMC5260151 28138005

[jcv212020-bib-0015] Leppert, B. , Havdahl, A. , Riglin, L. , Jones, H. J. , Zheng, J. , Davey Smith, G. , Tilling, K. , Thapar, A. , Reichborn‐ Kjennerud, T. , & Stergiakouli, E. (2019). Association of maternal neurodevelopmental risk alleles with early‐life exposures. JAMA Psychiatry, 76(8), 834–842. 10.1001/jamapsychiatry.2019.0774 31042271 PMC6495368

[jcv212020-bib-0016] Liew, Z. , Bach, C. C. , Asarnow, R. F. , Ritz, B. , & Olsen, J. (2016). Paracetamol use during pregnancy and attention and executive function in offspring at age 5 years. International Journal of Epidemiology, 45(6), 2009–2017. 10.1093/ije/dyw296 28031314

[jcv212020-bib-0017] Liew, Z. , Kioumourtzoglou, M.‐A. , Roberts, A. L. , O'Reilly, E. J. , Ascherio, A. , & Weisskopf, M. G. (2019). Use of negative control exposure analysis to evaluate confounding: An example of acetaminophen exposure and attention‐deficit/hyperactivity disorder in nurses' health study II. American Journal of Epidemiology, 188(4), 768–775. 30923825 10.1093/aje/kwy288 PMC6438812

[jcv212020-bib-0018] Liew, Z. , Ritz, B. , Rebordosa, C. , Lee, P.‐C. , & Olsen, J. (2014). Acetaminophen use during pregnancy, behavioral problems, and hyperkinetic disorders. JAMA Pediatrics, 168(4), 313–320. 24566677 10.1001/jamapediatrics.2013.4914

[jcv212020-bib-0019] Lupattelli, A. , Spigset, O. , Twigg, M. J. , Zagorodnikova, K. , Mårdby, A. C. , Moretti, M. E. , Drozd, M. , Panchaud, A. , Hämeen‐Anttila, K. , Rieutord, A. , Gjergja Juraski, R. , Odalovic, M. , Kennedy, D. , Rudolf, G. , Juch, H. , Passier, A. , Björnsdóttir, I. , & Nordeng, H. (2014). Medication use in pregnancy: A cross‐sectional, multinational web‐based study. BMJ Open, 4(2), e004365. 10.1136/bmjopen-2013-004365 PMC3927801 24534260

[jcv212020-bib-0020] Magnus, P. , Birke, C. , Vejrup, K. , Haugan, A. , Alsaker, E. , Daltveit, A. K. , et al. (2016). Cohort profile update: The Norwegian mother and child cohort study (MoBa). International Journal of Epidemiology, 45(2), 382–388. 27063603 10.1093/ije/dyw029

[jcv212020-bib-0021] Magnus, P. , Irgens, L. M. , Haug, K. , Nystad, W. , Skjaerven, R. , & Stoltenberg, C. (2008). Cohort profile: The Norwegian Mother and Child Cohort Study (MoBa). International Journal of Epidemiology, 35(5), 1146–1150. 10.1093/ije/dyl170 16926217

[jcv212020-bib-0022] Masarwa, R. , Levine, H. , Gorelik, E. , Reif, S. , Perlman, A. , & Matok, I. (2018). Prenatal exposure to acetaminophen and risk for attention deficit hyperactivity disorder and autistic spectrum disorder: A systematic review, meta‐analysis, and meta‐regression analysis of cohort studies. American Journal of Epidemiology, 187(8), 1817–1827. 29688261 10.1093/aje/kwy086

[jcv212020-bib-0023] Masarwa, R. , Platt, R. W. , & Filion, K. B. (2020). Acetaminophen use during pregnancy and the risk of attention deficit hyperactivity disorder: A causal association or bias? Paediatric & Perinatal Epidemiology, 34(3). 10.1111/ppe.12615 31916282

[jcv212020-bib-0024] Nilsen, R. M. , Vollset, S. E. , Gjessing, H. K. , Skjærven, R. , Melve, K. K. , Schreuder, P. , et al. (2009). Self‐selection and bias in a large prospective pregnancy cohort in Norway. Paediatric & Perinatal Epidemiology, 23(6), 597–608. 19840297 10.1111/j.1365-3016.2009.01062.x

[jcv212020-bib-0025] Oerbeck, B. , Overgaard, K. R. , Aspenes, S. T. , Pripp, A. H. , Mordre, M. , Aase, H. , Reichborn‐Kjennerud, T. , & Zeiner, P. (2017). ADHD, comorbid disorders and psychosocial functioning: How representative is a child cohort study? Findings from a national patient registry. BMC Psychiatry, 17(1), 23. 10.1186/s12888-017-1204-7 28095819 PMC5240379

[jcv212020-bib-0026] PRAC recommendations on signals . (2019). Retrieved from https://www.ema.europa.eu/en/documents/prac‐recommendation/prac‐recommendations‐signals‐adopted‐12‐15‐march‐2019‐prac‐meeting_en.pdf

[jcv212020-bib-0027] RStudio Team (2020). RStudio: Integrated development for R. RStudio, PBC, Boston, MA URL. http://www.rstudio.com/

[jcv212020-bib-0028] Saunders, G. R. B. , McGue, M. , & Malone, S. M. (2019). Sibling comparison designs: Addressing confounding bias with inclusion of measured confounders. Twin Research and Human Genetics, 22(5), 290–296. 10.1017/thg.2019.67 31559947 PMC7170177

[jcv212020-bib-0029] Sjölander, A. , Frisell, T. , Kuja‐Halkola, R. , Öberg, S. , & Zetterqvist, J. (2016). Carryover effects in sibling comparison designs. Epidemiology, 27(6), 852–858. 27488059 10.1097/EDE.0000000000000541

[jcv212020-bib-0030] Sölander, A. , & Zetterqvist, J. (2017). Confounders, mediators, or colliders what types of shared covariates does a sibling comparison design control for? Epidemiology, 28(4), 540–547. 28575894 10.1097/EDE.0000000000000649

[jcv212020-bib-0031] StataCorp (2015). Stata statistical software: Release 14. College Station, TX: StataCorp LP.

[jcv212020-bib-0032] Stergiakouli, E. , Thapar, A. , & Davey Smith, G. (2016). Association of acetaminophen use during pregnancy with behavioral problems in childhood evidence against confounding. JAMA Pediatrics, 170(10), 964–970. 27533796 10.1001/jamapediatrics.2016.1775 PMC5300094

[jcv212020-bib-0033] Tambs, K. , & Moum, T. (1993). How well can a few questionnaire items indicate anxiety and depression?. Acta Psychiatrica Scandinavica, 87(5), 364–367. 8517178 10.1111/j.1600-0447.1993.tb03388.x

[jcv212020-bib-0034] Thompson, J. M. D. , Waldie, K. E. , Wall, C. R. , Murphy, R. , Mitchell, E. A. , & the ABC study group (2014). Associations between Acetaminophen Use during Pregnancy and ADHD Symptoms Measured at Ages 7 and 11 Years. PloS One, 9(9). 10.1371/journal.pone.0108210 PMC4177119 25251831

[jcv212020-bib-0035] Trønnes, J. N. , Wood, M. , Lupattelli, A. , Ystrom, E. , & Nordeng, H. (2019). Prenatal paracetamol exposure and neurodevelopmental outcomes in preschhol‐aged children. Paediatric & Perinatal Epidemiology, 34(3), 247–256. 31448449 10.1111/ppe.12568 PMC8285062

[jcv212020-bib-0036] WHO Collaborating Centre for Drug Statistics Methodology . (2018). ATC/DDD Index 2019. Retrieved from https://www.whocc.no/atc_ddd_index/

[jcv212020-bib-0037] Wood, M. E. , Lapane, K. L. , van Gelder, M. M. H. J. , Rai, D. , & Nordeng, H. M. E. (2018). Making fair comparisons in pregnancy medication safety studies: An overview of advanced methods for confounding control. Pharmacoepidemiology and Drug Safety, 27(2), 140–147. 29044735 10.1002/pds.4336 PMC6646901

[jcv212020-bib-0038] World Health Organization (WHO) . (1993). The ICD‐10 Classification of Mental and Behavioural Disorders. Genève, Switzerland: World Health Organization.

[jcv212020-bib-0039] Ystrom, E. , Gustavson, K. , Brandlistuen, R. E. , Knudsen, G. P. , Magnus, P. , Susser, E. , Davey Smith, G. , Stoltenberg, C. , Surén, P. , Håberg, S. E. , Hornig, M. , Lipkin, W. I. , Nordeng, H. , & Reichborn‐Kjennerud, T. (2017). Prenatal exposure to Acetaminophen and risk of ADHD. Pediatrics, 140(5), e20163840. 10.1542/peds.2016-3840 PMC5654387 29084830

