# Supplementary material for: Acetaminophen use during pregnancy and offspring attention deficit hyperactivity disorder – a longitudinal sibling control study
Source: JCPP Adv. 2021 Jun 22;1(2):e12020. doi: 10.1002/jcv2.12020 (PMC10242945; doi:10.1002/jcv2.12020)
Supplement: Supplementary file 1 — Supporting Information [file JCV2-1-e12020-s001.docx]

**Supporting information for:**

*Acetaminophen use during pregnancy and offspring attention deficit hyperactivity disorder – a longitudinal sibling control study*

1. **Appendix S1. Supporting Methods:**
   1. Missing data
   2. Combination products
   3. Sources of bias
      1.3.1. Carryover effects

1.3.2. Non-shared confounders

1.3.3. Measurement error

1. **Appendix S2. Supporting Results:**
   1. Missing data

Table S1

Table S2

- 1. Propensity scores: Table S3
  2. Numbers of discordant siblings
  3. Sources of bias
     1. Carryover effects
     2. Non-shared confounders

Table S4

- - 1. Measurement error

Table S5

Fig S1

Fig S2

**Appendix S1. Supporting methods:**

- 1. **Missing data.**

A total of 1,410 children lacked information about number of days exposed to acetaminophen. Only children from families with information about number of days from two or more children were included in analyses, and 2,477 children were therefore excluded because they lacked exposure information themselves or because their siblings did.

Also, 5,165 children lacked information on covariates, and therefore excluded from analyses.

Multiple imputation (MI), which is often the preferred choice for dealing with missing data (Schafer & Graham, 2002) was considered, but not performed in the main analyses. This was because the main analyses were bootstrapped with 1,000 replications. Multiplying this with 20-50 imputed data sets would have created a very complex data structure.

Potential bias due to missing data was therefore investigated.

To examine potential bias due to missing values on number of days, we performed sensitivity analyses where the missing values on number of days were redistributed (recoded) to one of the different exposure categories (1-7 days in one analysis, 8-28 days in the next analysis, and 29 days or more in the third analysis). This was done to compare association estimates from analyses where only families with information about number of days for at least to children were included (as in the main analyses in the manuscript) to estimates from analyses where it was assumed that children with missing information about number of days mainly belong to one of the three exposure groups (1-7 days, 8-28 days, or 29 days or more).

Cox regression analyses were performed (without bootstrapping) four times: Frist, only those who had information about number of days acetaminophen exposure for at least two children were included (as in the main analyses in the manuscript). Then three sets of the same analyses were performed with missing information on number of days recoded to 1) 1-7 days, 2) 8-28 days, and 3) 29 days or more. All analyses were adjusted for birth year, and for dependency in the data by using the cluster option in Stata.

Results are presented below in Supporting results, and in Table S1.

Another set of sensitivity analyses were performed to examine potential bias due to excluding cases with missing values on covariates. These analyses were run without bootstrapping. MI of covariates was performed, where 20 imputed data sets were generated using MICE (multiple imputation by chained equations (van Buuren, 2018)), and the PMM (predictive mean matching) method (van Buuren, 2018). Analyses adjusted for observed covariates were then performed only on the complete data (as in the main analyses in the manuscript) and then on the MI data. This was done to compare the magnitude of associations between exposure to acetaminophen and ADHD diagnosis in MI versus complete case analyses. Results are presented below in Supporting results, and in Table S2.

- 1. **Combination products:**

Some products contain acetaminophen in addition to other active ingredients. The following medications were examined in the current sample: N02B E51, N02AJ13, N02AJ06, N02BE75, and N02AA59. Only the latter was used during pregnancy by mothers of siblings in the MoBa. N02AA59 was previously used for combinations of codeine + acetaminophen, as well as for codeine + acetylsalicylic acid, codeine + ibuprofen, and codeine + other non-opioid analgesics. 1.6% of the children were exposed to N02AA59 during pregnancy in the current sample, and 76% of those were also exposed to N02BE0. Hence, 0.4% of the children in the current sample were exposed to N02AA59, but not to N02BE0. These were not included in the analyses.

- 1. **Sources of bias:**
     1. **Carryover effects**

Results from sibling control studies may be biased if the exposure and outcome of one sibling are not independent of the exposure and outcome of the other sibling (Sjolander, Frisell, Kuja-Halkola, Oberg, & Zetterqvist, 2016). Not all such potential carryover effects are possible to examine. However, inspired by a similar example by Sjölander et al. (2016) we have examined the following potential carryover effect:

If one sibling develops ADHD, parents may be affected in several ways. They may be more exhausted and thus prone to inflammations or pain conditions, or they may develop a lower threshold for using painkillers for such conditions because of a demanding everyday life. We examined this as suggested by a similar example in Sjölander et al. (2016). We performed a negative binomial regression analysis where number of days maternal acetaminophen use in the second pregnancy (continuously measured) was regressed on the ADHD status of the first sibling, controlled for number of days acetaminophen use in the first pregnancy. Sibling three and four from families participating with more than two children were not included in these analyses.

The results are presented below in Supporting Results.

- - 1. **Non-shared confounders**

The sibling control design controls for unobserved confounding factors shared between siblings. However, confounding factors not shared between siblings may introduce bias in such analyses. See Frisell, Oberg, Kuja-Halkola, & Sjolander (2012) for a discussion of this. Frisell at al. (2012) point out that the sibling control design is useful in situations where the confounding factors are more shared between siblings than the exposure is, and that the design may introduce bias when the exposure is more shared between siblings than the confounders are.

We are not able to examine the degree of similarity between siblings regarding unobserved confounders. However, as suggested by Frisell et al. (2012), we have compared the sibling correlation in our main exposure (acetaminophen use 29 days or more) to the sibling correlations in several observed confounders. Important unobserved confounders for the association between acetaminophen use and ADHD in the child may be family socioeconomic position and maternal health and health behaviors. Maternal education, maternal smoking during pregnancy, maternal BMI before pregnancy, and a history of mental problems were used as proxies of the latent (unobserved) socioeconomic and health related confounders. Tetrachoric correlations were calculated for the exposure (acetaminophen use 29 days or more “yes” versus “no”) and for dichotomous proxies of unobserved confounding factors, while Pearson’s correlations were calculated for continuous proxies of unobserved confounders.

The results are presented below in Supporting Results and in Table S4.

- - 1. **Measurement error**

A third source of bias in sibling control studies is measurement error (Frisell et al., 2012). Unreliability in measures of the exposure can attenuate within-family estimates (i.e. estimates of differences between siblings) more than the estimates from analyses without sibling control. Hence, even in the absence of unobserved family confounding factors, the association between long-term acetaminophen exposure and ADHD may be attenuated in the sibling control model relative to the model without sibling control. Measurement error may thus lead to a false interpretation of observed associations being due to unobserved confounding factors. As maternal report of acetaminophen exposure may be prone to measurement error, this bias may be particularly relevant in the current study.

We therefore performed several sensitivity analyses to examine this. First, we used simulation analyses to investigate how much the association between long-term exposure to acetaminophen and ADHD would be reduced in the sibling control model compared to the model without sibling control, only because of measurement error. The R script provided by Frisell et al. (2012) was used to simulate data for different levels of unreliability. Frisell et al. (2012) operationalize reliability as sensitivity (i.e. the degree to which a true positive value is detected by the measure) and specificity (i.e. the degree to which a true negative value is identified correctly as negative). Sensitivity and specificity are 1.00 when reliability is perfect.

The script was modified to fit with the parameters in the current study. The prevalence of exposure (long-term acetaminophen exposure) was set to 2%, and the prevalence of the outcome was set to 5.0% (as in the group exposed for 29 days or more). The tetrachoric correlation between the siblings’ exposures was set to Rho = 0.71. True OR was varied between 5, 10, and 15. N was set to 1 000 000, as in Frisell et al. (2012). A high N is beneficial to minimize effects of random sampling on the results.

The results of these simulations are presented below in Supporting Results and in Table S5.

Cox regression analyses were also performed excluding acetaminophen use for indications reported both in the last part of pregnancy and in the months after birth. Results are presented below in Fig S1.

We also performed Cox regression analyses excluding children of mothers that had taken acetaminophen as well as other medications for the same indications (excluding 1,126 children from analyses). Results are presented below in Fig S2.

1. **Appendix S2. Supporting results:**

**2.1. Missing data**

*Bias from missing information on number of days:* Results from analyses with missing values on number of days recoded to the different exposure groups, are shown in Table S1. The table shows that if children with missing information about number of days in reality mainly belonged to the first exposure category (1-7 days), our main analyses where these children and their siblings were excluded, over-estimated associations between long-term acetaminophen exposure and ADHD somewhat. The same was true if children with missing information about number of days in reality mainly belonged to the second category (8-28 days). If they mainly belonged to the third category (29 days or more), our main analyses were practically unbiased. These sensitivity analyses thus suggested that our main results regarding long-term exposure were unbiased, or slightly over-estimated, due to missing information about number of days for some children. Regardless of which category these children were redistributed to, the does-response pattern from the main analyses was present.

Because bootstrapping was not performed, the CIs for the main analyses in Table S1 differs somewhat from the CIs of the unadjusted results in the main manuscript.

The results regarding bias due to missing values on covariates are presented in Table S2. The table shows results from complete case analyses versus results from MI of covariates. These results suggested that if there is a bias due to excluding children with missing values on covariates in the main analyses, it might be towards over-estimation of the association between long-term use and ADHD. However, complete case and MI results are similar. Bootstrapping was not performed.

**Table S1**. Results from main analysis, versus from three analyses where missing values on number of days were redistributed to different exposure categories.

|  | Main analysis | Redistributed to exposure category  1-7 days ^a)^ | Redistributed to exposure category 8-28 days ^b)^ | Redistributed to exposure category 29 days or more ^c)^ |
| --- | --- | --- | --- | --- |
| Exposure group | HR  95% CI | HR  95% CI | HR  95% CI | HR  95% CI |
| Unexposed | Reference | Reference | Reference | Reference |
| 1-7 days | 1.03  0.83-1.26 | 1.15  0.96-1.39 | 1.01  0.82-1.23 | 1.00  0.82-1.23 |
| 8-28 days | 1.35  1.04-1.75 | 1.34  1.04-1.73 | 1.55  1.25-1.93 | 1.34  1.04-1.28 |
| 29 days or more | 1.99  1.26-3.14 | 1.82  1.16-2.87 | 1.83  1.16-2.88 | 2.01  1.54-2.63 |

^Notes: Main analysis: excluding families with less than two siblings with information on number of days. HR = hazard ratio, SE = standard error. Analyses were adjusted for birth year and stratified on indication for use. a) Missing values on number of days were recoded to 1-7 days, b) Missing values recoded to 8-28 days, c) Missing values recoded to 29 days or more. Standard errors were adjusted for dependency between siblings’ data with the cluster option.^

**Table S2**. Complete case and multiple imputation analyses.

|  | CC | MI |
| --- | --- | --- |
|  | HR  95% CI | HR  95% CI |
| Unexposed | Reference | Reference |
| 1-7 days | 1.04  0.87-1.27 | 1.11  0.93-1.31 |
| 8-28 days | 1.42  1.11-1.82 | 1.48  1.19-1.86 |
| 29 days or more | 2.30  1.50-3.53 | 2.08  1.38-3.13 |

^Notes: HR = Hazard ratio, CI = confidence interval, CC = complete case analysis, MI= multiple imputation with 20 imputed data sets. Analyses were adjusted for child’s birth year, maternal age, education, parity, alcohol use during pregnancy, smoking during pregnancy, symptoms of anxiety and depression. Standard errors were adjusted for dependency between siblings’ data with the cluster option.^

**2.2. Propensity scores:**

Distributions of propensity scores in the four exposure groups (i.e. unexposed, exposed 1-7 days, 8-28 days, and 29 days or more) are shown in Table S3.

**Table S3.** Distribution of propensity scores in the four exposure groups.

|  | Propensity scores for being unexposed | Propensity scores for exposure 1-7 days | Propensity scores for  exposure 8-28 days | Propensity scores for exposure 29 days or more |
| --- | --- | --- | --- | --- |
| Unexposed:  *Min*  *Mean*  *Max*  *Standard deviation* | 0.19  0.63  0.87  0.12 | 0.13  0.32  0.40  0.07 | 0.00  0.04  0.23  0.05 | 0.00  0.00  0.38  0.02 |
| Exposed 1-7 days:  *Min*  *Mean*  *Max*  *Standard deviation* | 0.11  0.48  0.86  0.13 | 0.13  0.36  0.40  0.05 | 0.00  0.12  0.23  0.08 | 0.00  0.04  0.64  0.08 |
| Exposed 8-28 days:  *Min*  *Mean*  *Max*  *Standard deviation* | 0.07  0.39  0.80  0.14 | 0.08  0.34  0.40  0.07 | 0.00  0.16  0.23  0.07 | 0.00  0.12  0.77  0.15 |
| Exposed 29 days or more:  *Min*  *Mean*  *Max*  *Standard deviation* | 0.06  0.28  0.74  0.14 | 0.07  0.28  0.40  0.09 | 0.00  0.17  0.23  0.06 | 0.00  0.27  0.81  0.22 |

**2.3. Number of discordant siblings:**

Only discordant siblings contribute with information in the sibling design. In the current sample, 5,327 mothers participated with children discordant on the exposure (4,885 of these participated with two children, the rest with three or four children). Also, 623 mothers participated with children discordant on the outcome (571 of these participated with two children, the rest with three or four children). A total of 306 mothers participated with children that were discordant on exposure as well as on outcome (274 of these with two children, 32 with 3 or 4 children). 380 mothers participated with children discordant on the exposure for 29 days or more, 34 of these also had children discordant on the outcome (30 of these had two children, while 4 had 3 children).

These numbers show that statistical power to detect within effects was relatively low. Hence, these results should be interpreted with caution.

**2.4. Sources of bias:**

**2.4.1. Carry-over effects**

The results from the negative binomial regression analysis where the number of days sibling two was exposed to acetaminophen was predicted by the first sibling’s ADHD diagnosis, controlled for the first sibling’s number of days exposed to acetaminophen, were the following: b = 0.10, p =0.39, SE=0.12, 95% CI = -0.13;0.34.

This showed that when sibling one had an ADHD diagnosis, sibling two was exposed to acetaminophen for exp (0.10) = 1.1 days more than when sibling one did not have an ADHD diagnosis. This difference was not statistically significant (95% CI ranged from 0.9 days less to 1.4 days more exposed). Hence, this did not provide evidence for carryover effects from sibling one’s ADHD diagnosis to sibling two’s acetaminophen exposure.

**2.4.2. Non-shared confounders.**

As Table S4 shows, several of the observed confounders showed higher correlation between siblings than did the exposure.

**Table S4.** Correlations between siblings regarding the main exposure and several observed confounding factors, used as proxies for unobserved confounders.

|  | Exposure: Acetaminophen >= 29 days (yes/no) | Dichotomous proxy for maternal mental health:  A history of mental disorders (yes/no) | Dichotomous proxy for maternal health and health behavior: Smoking before pregnancy (yes/no) | Continuous proxy for maternal health and health behavior: BMI before pregnancy | Continuous proxy for family socioeconomic status: Maternal education. |
| --- | --- | --- | --- | --- | --- |
| Correlation | 0.71*** | 0.82*** | 0.92*** | 0.91*** | 0.86*** |

^Notes: Tetrachoric correlations were estimated for dichotomous variables, and Pearson’s correlations for continuous variables. *** p < 0.001^

**2.4.3. Measurement error**

**Simulation analyses:**

Results from the simulation analyses are shown in Table S5. The first two columns show degree of unreliability – lower numbers represent less reliability. Column four and five show estimated associations (OR = Odds ratios) between long-term acetaminophen exposure and ADHD in the models without sibling control (column four) and the model with sibling control (column 5) at different levels of unreliability of the measure and at different levels of true associations between acetaminophen exposure and ADHD (column three). The simulated scenarios did not contain any unobserved confounding. Hence, attenuation of estimates was entirely due to measurement error.

The table shows that the estimated associations between long-term acetaminophen exposures from a model without sibling control were substantially attenuated as sensitivity and specificity were reduced. The observed associations from models with sibling control were even more attenuated. However, the estimates from the sibling control model were 87-98% of the estimates from the model without sibling control. The results from the main analyses in the current study showed that the estimate of the association between long-term acetaminophen exposure and ADHD from the sibling control model was 52% of the estimate from the model without sibling control. Hence, the simulations did not suggest that the attenuation observed in the current study in the sibling control model could be entirely (or mostly) explained by bias due to measurement error.

**Table S5.** Results from simulation analyses of the effects of different degree of unreliability on association estimates from analyses with versus without sibling control.

| Sensitivity | Specificity | True OR | Observed OR in model without sibling control | Observed OR in model with sibling control | Observed OR with sibling control / observed OR without sibling control |
| --- | --- | --- | --- | --- | --- |
| 0.8 | 0.8 | 5.00 | 1.23 | 1.15 | 0.93 |
| 0.8 | 0.8 | 10.00 | 1.45 | 1.30 | 0.90 |
| 0.8 | 0.8 | 15.00 | 1.62 | 1.41 | 0.87 |
|  |  |  |  |  |  |
| 0.7 | 0.7 | 5.00 | 1.13 | 1.09 | 0.96 |
| 0.7 | 0.7 | 10.00 | 1.22 | 1.15 | 0.94 |
| 0.7 | 0.7 | 15.00 | 1.32 | 1.22 | 0.92 |
|  |  |  |  |  |  |
| 0.6 | 0.6 | 5.00 | 1.04 | 1.02 | 0.98 |
| 0.6 | 0.6 | 10.00 | 1.11 | 1.08 | 0.97 |
| 0.6 | 0.6 | 15.00 | 1.14 | 1.10 | 0.96 |

^Notes: OR = Odds Ratio, sensitivity = proportion of true positives (i.e. those who have used acetaminophen for 29 days or more) correctly identified by the measure. Specificity = proportion of true negatives (i.e. those who have not used acetaminophen for 29 days or more) correctly identified by the measure. The simulated scenarios did not contain any unobserved confounding.^

**Cox regression analyses – measurement error:**

To further examine bias due to measurement error, Cox regression analyses were performed excluding acetaminophen use for indications reported both in the last part of pregnancy and in the months after birth. The results are shown in Fig S1. Next, we performed Cox regression analyses excluding children of mothers that had used other medications in addition to acetaminophen. The results are shown in Fig S2.

**Fig S1:** **Results from Cox regression analyses excluding acetaminophen use for indications reported both in the last part of pregnancy and in the months after birth.**


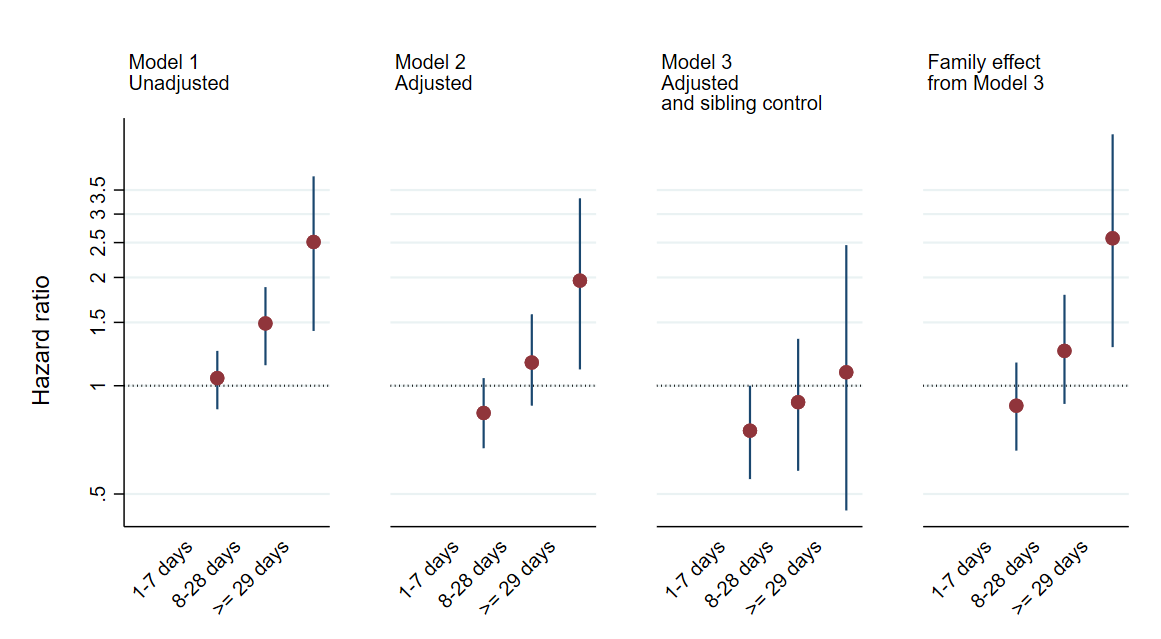
^
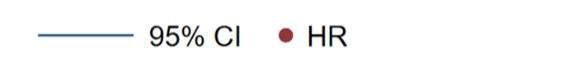
^

^Notes: HR = Hazard Ratio, C.I. = bias corrected bootstrap 95% confidence interval (1,000 replications), Model 2 was adjusted for propensity scores for number of days exposed as well as for maternal education and parity. Model 3 was adjusted for propensity scores for number of days exposed. The rightmost column shows the family effect in the sibling control model.^

**Fig S2:** **Results from Cox regression analyses excluding children of mothers who had taken other medication in addition to acetaminophen.**

^
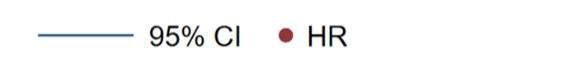
^
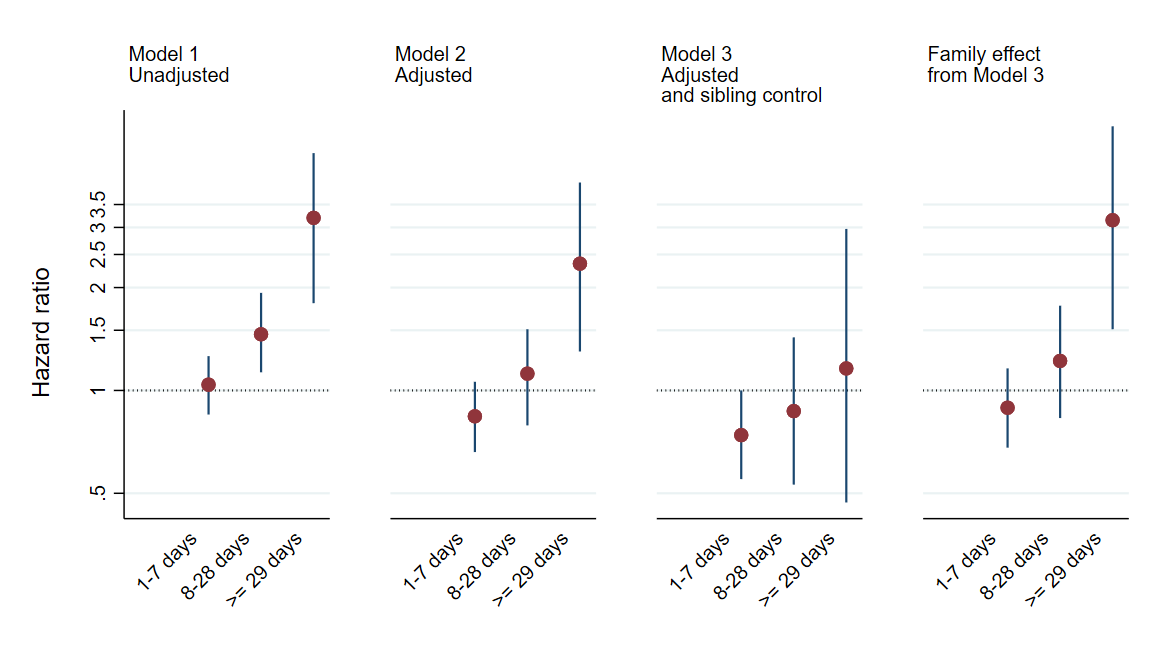


^Notes: HR = Hazard Ratio, C.I. = bias corrected bootstrap 95% confidence interval (1,000 replications), Model 2 was adjusted for propensity scores for number of days exposed as well as for maternal education and parity. Model 3 was adjusted for propensity scores for number of days exposed. The rightmost column shows the family effect in the sibling control model.^

**References**

Frisell, T., Oberg, S., Kuja-Halkola, R., & Sjolander, A. (2012). Sibling comparison designs: bias from non-shared confounders and measurement error. *Epidemiology, 23*(5), 713-720. doi:10.1097/EDE.0b013e31825fa230

Schafer, J. L., & Graham, J. W. (2002). Missing data: Our view of the state of the art. *Psychological Methods, 7*(2), 147-177.

Sjolander, A., Frisell, T., Kuja-Halkola, R., Oberg, S., & Zetterqvist, J. (2016). Carryover Effects in Sibling Comparison Designs. *Epidemiology, 27*(6), 852-858. doi:10.1097/EDE.0000000000000541

van Buuren, S. (2018). *Flexible imputation of missing data*: Chapman & Hall/CRC.
